# SARS-Coronavirus Replication/Transcription Complexes Are Membrane-Protected and Need a Host Factor for Activity *In Vitro*

**DOI:** 10.1371/journal.ppat.1000054

**Published:** 2008-05-02

**Authors:** Martijn J. van Hemert, Sjoerd H. E. van den Worm, Kèvin Knoops, A. Mieke Mommaas, Alexander E. Gorbalenya, Eric J. Snijder

**Affiliations:** 1 Molecular Virology Laboratory, Department of Medical Microbiology, Leiden University Medical Center, Leiden, The Netherlands; 2 Section Electron Microscopy, Department of Molecular Cell Biology, Leiden University Medical Center, Leiden, The Netherlands; University of North Carolina, United States of America

## Abstract

SARS-coronavirus (SARS-CoV) replication and transcription are mediated by a replication/transcription complex (RTC) of which virus-encoded, non-structural proteins (nsps) are the primary constituents. The 16 SARS-CoV nsps are produced by autoprocessing of two large precursor polyproteins. The RTC is believed to be associated with characteristic virus-induced double-membrane structures in the cytoplasm of SARS-CoV-infected cells. To investigate the link between these structures and viral RNA synthesis, and to dissect RTC organization and function, we isolated active RTCs from infected cells and used them to develop the first robust assay for their *in vitro* activity. The synthesis of genomic RNA and all eight subgenomic mRNAs was faithfully reproduced by the RTC in this *in vitro* system. Mainly positive-strand RNAs were synthesized and protein synthesis was not required for RTC activity *in vitro*. All RTC activity, enzymatic and putative membrane-spanning nsps, and viral RNA cosedimented with heavy membrane structures. Furthermore, the pelleted RTC required the addition of a cytoplasmic host factor for reconstitution of its *in vitro* activity. Newly synthesized subgenomic RNA appeared to be released, while genomic RNA remained predominantly associated with the RTC-containing fraction. RTC activity was destroyed by detergent treatment, suggesting an important role for membranes. The RTC appeared to be protected by membranes, as newly synthesized viral RNA and several replicase/transcriptase subunits were protease- and nuclease-resistant and became susceptible to degradation only upon addition of a non-ionic detergent. Our data establish a vital functional dependence of SARS-CoV RNA synthesis on virus-induced membrane structures.

## Author Summary

The SARS-coronavirus (SARS-CoV), which causes the life-threatening severe acute respiratory syndrome, replicates in the cytoplasm of infected host cells. A critical early step in the SARS-CoV life cycle is the formation of a replication/transcription complex (RTC) that drives viral genome replication and subgenomic mRNA synthesis. Virus-encoded enzymes form the core of this RTC, which is believed to be associated with characteristic virus-induced membrane structures derived from modified host cell membranes. To investigate the connection between these membrane structures and SARS-CoV RNA synthesis, and to characterize RTC composition and function, we isolated these complexes and developed the first *in vitro* assay to study their activity. SARS-CoV genomic RNA and all eight subgenomic mRNAs were synthesized in this *in vitro* reaction. By centrifugation, RTC activity could be isolated from the cytoplasm, together with membrane structures, viral enzymes, and RNA. The activity of these isolated RTCs was dependent on a cytoplasmic host factor. RTC activity was destroyed by detergent treatment, suggesting a critical role for membranes that appeared to protect the complex against protease and nuclease digestion. Our data establish a functional connection between viral RNA synthesis and intracellular membranes and show that host factors play a crucial role in SARS-CoV RNA synthesis.

## Introduction

Following infection and genome translation, positive-strand RNA (+RNA) viruses establish a cytoplasmic enzyme complex that directs the amplification and expression of their genome. The viral RNA-dependent RNA polymerase (RdRp) is the central enzyme of this ‘replication/transcription complex’ (RTC), but it also may include other viral non-structural proteins (nsps) and host factors that cooperate to synthesize viral RNA. Over the past decade, it has become clear that +RNA virus RTCs are invariably associated with virus-induced membrane structures, which are poorly characterized but presumably provide a framework for RNA synthesis by facilitating the concentration and cooperation of viral macromolecules on a dedicated membrane surface. They may also protect the viral RNA from nucleases in the cytoplasm of the host cell, aid in shielding the double-stranded RNA intermediates of virus replication from the host cell's innate immune system, or contribute to the coordination of the viral life cycle in time and space. These membrane-bound RTCs are the molecular machines that drive the RNA synthesis and evolution of +RNA viruses. Clearly, unraveling their structure and function will be critical to understand the biochemistry of +RNA virus replication and develop novel antiviral control strategies.

The RTC of coronaviruses, including that of SARS-coronavirus (SARS-CoV), the causative agent of the life-threatening severe acute respiratory syndrome (for a review, see reference [1]), stands out for a number of reasons. First, at 27–32 kb, the polycistronic coronavirus genome is by far the largest genome among currently known RNA viruses [2]. Second, the viral RNA-synthesizing machinery not only amplifies the genome, but also directs the synthesis of a set of subgenomic (sg) mRNAs (eight in the case of SARS-CoV; RNA2 to RNA9) to express the viral accessory and structural protein genes. The latter are produced from a corresponding set of subgenome-length negative strand RNAs, which derive from discontinuous negative-strand RNA synthesis [3],[4]. Third, the viral replicase/transcriptase (which will be referred to as “replicase” for brevity) is of unprecedented size and complexity [5],[6]. It is produced by translation of the partly overlapping open reading frames (ORF) 1a and 1ab, with expression of the latter requiring a -1 ribosomal frameshift near the end of ORF1a. In this manner, SARS-CoV genome translation yields the large replicase polyproteins pp1a (4,382 aa) and pp1ab (7,073 aa). Extensive autoproteolytic processing, mediated by two ORF1a-encoded protease domains [7]–[10], ultimately generates 16 nsps [5],[6],[11],[12]. These include key replicative enzymes (*e.g.* the nsp12-RdRp [13], and the nsp13-helicase [14]), a variety of subunits containing presumed accessory functions for viral RNA synthesis (*e.g.* the nsp8-primase [15],[16], nsp14-exoribonuclease [17],[18], and nsp15-endoribonuclease NendoU [19]–[22]) and several predicted multi-spanning membrane proteins (nsp3, nsp4 and nsp6; [23],[24]) that presumably modify cellular endomembranes and target the RTC to this scaffold.

Immunofluorescence microscopy previously revealed that newly synthesized SARS-CoV RNA and several nsps colocalize in perinuclear foci in SARS-CoV-infected cells [8], [14], [25]–[27]. Electron microscopy established the presence of typical paired membranes, membrane whorls, and double-membrane vesicles (DMVs), which labeled for nsps [26]–[29] and viral RNA [27] and were therefore proposed to carry the SARS-CoV RTC. The endoplasmic reticulum (ER) was identified as the most likely membrane donor [26] and recent electron tomography studies indeed revealed a network of SARS-CoV-induced membrane structures that is continuous with this organelle (Knoops et al., in preparation). In the past four years, substantial progress has been made in the characterization of individual replicase subunits using enzymatic assays, reverse and classical genetics, bioinformatics and structural studies. However, the composition and mechanistics of the native ribonucleoprotein complexes, in which these different components interact to drive coronavirus replication and transcription, have remained completely uncharacterized thus far. We therefore set out to isolate active RTCs from SARS-CoV-infected cells and used those to develop an *in vitro* system that faithfully reproduced the synthesis of both genomic and sg RNAs, mainly of positive polarity. RTC activity cosedimented with newly synthesized viral RNA and several replicase subunits in a dense membrane fraction containing structures that could be labeled for nsp3 and nsp4. The *in vitro* activity of the pelleted RTC depended on the presence of a cytoplasmic host factor. Furthermore, RTC activity was destroyed by addition of (non-ionic) detergents, which also released replicase subunits and (mainly) sg RNA from the membrane fraction. Protease and nuclease protection experiments indicated that viral RNA and nsps were protected by membranes, thus further substantiating the functional connection between SARS-CoV RNA synthesis and virus-induced membrane structures that appear to be essential for RTC activity.

## Results

### Isolation of active SARS-CoV RTCs

**Figure 1:**
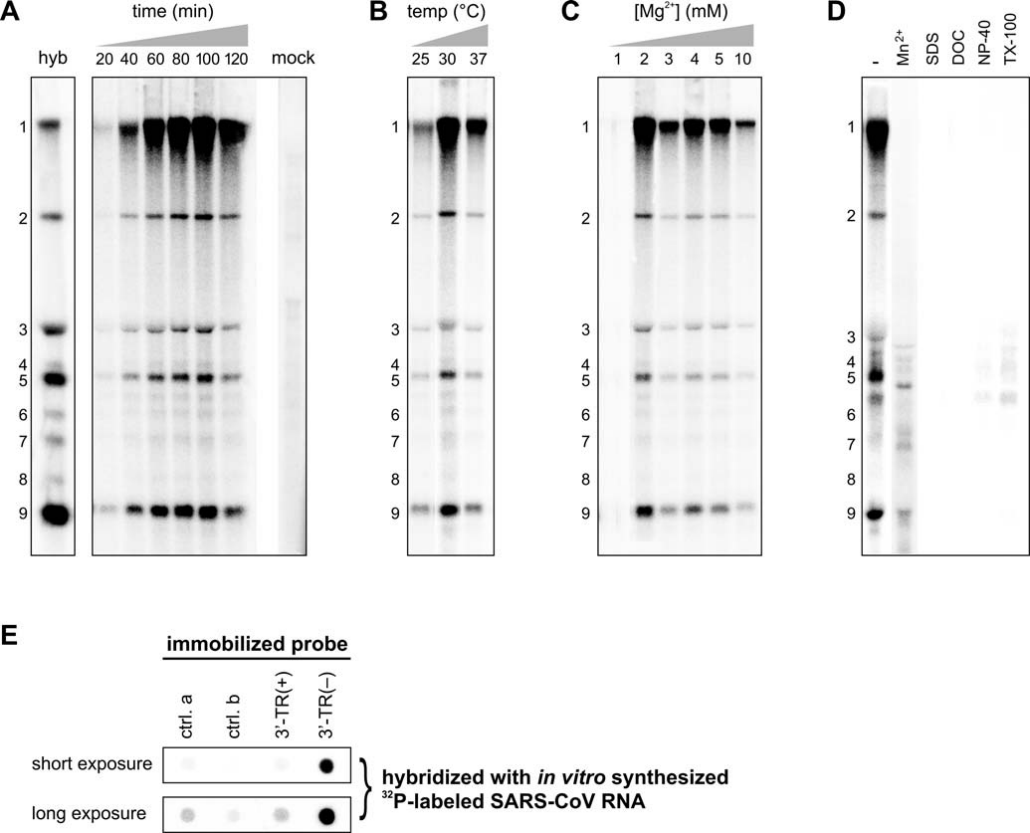
*In vitro* RNA synthesizing activity of SARS-CoV RTCs. Incorporation of [α-^32^P]CTP into viral RNA in IVRAs with PNS from SARS-CoV- or mock-infected cells was analyzed by denaturing formaldehyde-agarose gel electrophoresis, followed by PhosphorImager analysis. To optimize reaction conditions, the reaction time (A), temperature (B) and Mg^2+^ concentration (C) were varied, as indicated above the lanes. Except for the parameter that was varied, standardized conditions were used, as described in “Materials and Methods”. The “mock” lane contains the reaction products from a standard 100-min IVRA performed with a PNS from mock-infected cells. (D) IVRAs with 2 mM Mn^2+^ instead of Mg^2+^ or with 0.1% SDS, 0.03% DOC, 0.01% NP-40, or 0.01% TX-100 added, as indicated above the lanes. The position of RNAs 1-9 are indicated and were determined by including a lane (hyb) with RNA isolated from SARS-CoV-infected cells that was hybridized with a probe complementary to the 3′ end of all viral RNAs. The seemingly different ratios between genomic and sgRNA, when IVRA results are compared with the hybridization data, are explained by the fact that the former reflect the (size-dependent) incorporation of label whereas the latter show molar abundance. The molar ratios of IVRA products, calculated using the C-content of each RNA species, are similar to those in infected cells. (E) Analysis of the polarity of *in vitro* synthesized SARS-CoV RNA. A membrane containing immobilized SARS-CoV RNA probes of positive (3′-TR(+)) or negative polarity (3′-TR(-)) was hybridized with the ^32^P-labeled RNA products of an IVRA. RNA probes derived from the equine arteritis virus genome (ctrl. a) or its complement (ctrl. b) were included as negative controls. See Materials and Methods for probe details. A short and a long exposure of the same hybridization are shown.

In order to characterize isolated SARS-CoV RTCs, we developed an *in vitro* RNA synthesis assay (IVRA) to study their activity *in vitro*. In this reaction, the incorporation of [α-^32^P]CTP into viral RNA was analyzed in a mixture containing NTPs, Mg^2+^, an ATP-regenerating system, and an inhibitor of cellular transcription (Actinomycin D). The RTC activity in cytoplasmic extracts prepared from SARS-CoV-infected Vero-E6 cells produced a set of ^32^P-labeled RNA molecules with sizes corresponding to those of the SARS-CoV genome and all eight sg RNAs (Fig. 1). These products were not detected when using mock-infected cell lysates (Fig. 1A, mock), demonstrating that SARS-CoV RTC activity was responsible for their synthesis. Reaction conditions were optimized by varying several parameters, including the composition of the reaction mixture, incubation time, temperature, and the Mg^2+^ concentration (Fig. 1 and data not shown). In a time course experiment, *in vitro* synthesized viral RNA accumulated up to 100 min into the reaction (Fig. 1A), after which a decrease was observed, probably due to declining RTC activity in combination with continued RNA degradation by cellular nucleases. The optimal reaction temperature was 30°C (Fig. 1B). RTC activity was strongly dependent on the Mg^2+^ concentration and was maximal when 2 mM of Mg^2+^ was added to the reaction (Fig. 1C). Manganese could not replace Mg^2+^, as IVRAs containing Mn^2+^ only yielded a ladder of small radiolabeled RNA molecules with aberrant sizes (Fig. 1D), suggesting an effect on RdRp processivity. Addition of ionic (SDS and deoxycholate (DOC)) or non-ionic detergents (Nonidet P40 (NP-40) and Triton X-100 (TX-100)) to the post-nuclear supernatant (PNS) prior to the IVRA completely abolished the accumulation of radiolabeled viral RNA, suggesting that the integrity of membranes is an important factor for SARS-CoV RTC activity (Fig. 1D).

To determine the polarity of the *in vitro* produced RNAs, the ^32^P-labeled products of an IVRA were hybridized to a membrane containing immobilized RNA probes specific for SARS-CoV positive- or negative-stranded RNA (Fig. 1E). A strong hybridization with the positive strand-specific probe was observed, demonstrating that the RTC mainly synthesized RNA of positive polarity *in vitro*. After longer exposure times, a small quantity of radiolabeled material hybridizing to the negative strand-specific probe became visible, but a similar signal was observed with the negative control RNA (Fig. 1E). This indicated that the quantity of *in vitro* synthesized negative-stranded RNA was very small (less than 2% of the total RNA), which is in line with the large excess of positive over negative strands that is commonly observed *in vivo.*

### Protein synthesis is not required for RTC activity *in vitro*

**Figure 2:**
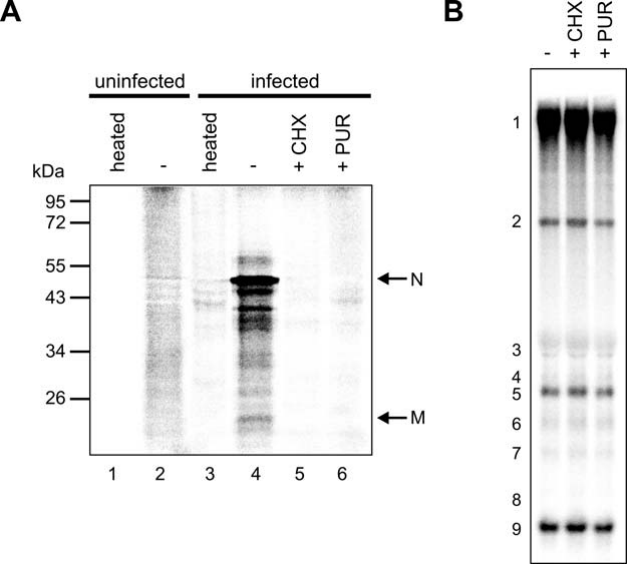
Protein synthesis is not required for RTC activity *in vitro*. (A) Incorporation of ^35^S-labeled amino acids into proteins during a 100-min IVRA. Proteins were separated by SDS-PAGE and incorporation of radiolabel was visualized by phosphorimaging of the dried gel. PNS from either uninfected or SARS-CoV-infected cells was used, which was either untreated (−) or heated to 96°C prior to the reaction, as indicated above the lanes. Reactions were performed in the absence (−) or presence of the translation inhibitors cycloheximide (+CHX) or puromycin (+PUR). The positions of protein size markers are indicated on the left and the arrows on the right indicate the positions of polypeptides with sizes matching those of SARS-CoV membrane (M) and nucleocapsid (N) proteins. (B) The effect of translation inhibitors on *in vitro* RTC activity. IVRAs were performed in the absence (−) or presence of cycloheximide (+CHX) or puromycin (+PUR) as indicated above the lanes. Reaction products were analyzed as described in the legend to Fig. 1.

To assess whether protein synthesis occurred during IVRAs, we determined whether ^35^S-labeled amino acids were incorporated into proteins during a 100-min reaction. When using the PNS of uninfected cells, SDS-PAGE revealed a smear of ^35^S-labeled material (Fig. 2A, lane 2). These products were absent when the PNS was heated to 96°C for 5 min prior to the assay (Fig. 2A, lane 1), suggesting they resulted from translation under IVRA conditions. When using the PNS of SARS-CoV-infected cells, we observed incorporation of radiolabel also into a set of discrete polypeptides (Fig. 2A, lane 4), including species with sizes matching those of the SARS-CoV membrane (M) and nucleocapsid (N) proteins. This was likely due to the fact that the lysate contained large amounts of the sg mRNAs encoding these proteins, possibly in combination with the virus-induced shut-off of host cell translation [30]. Protein synthesis was completely inhibited when the translation inhibitors cycloheximide or puromycin were present during the IVRA (Fig. 2A, lanes 5 and 6), but this did not affect *in vitro* RTC activity since the quantity of radiolabeled RNA products was unchanged (Fig. 2B).

### The activity of isolated RTCs depends on a cytoplasmic host factor

**Figure 3:**
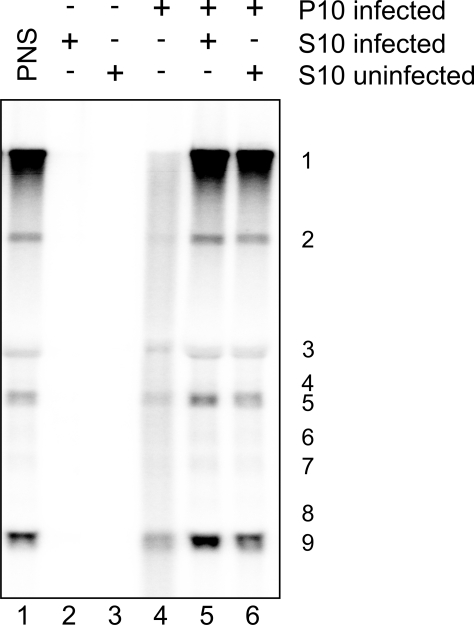
The activity of isolated RTCs is dependent on a cytoplasmic host factor. The PNS of SARS-CoV-infected or uninfected cells was subjected to differential centrifugation after which IVRAs were performed using the PNS (lane 1), 10,000×g pellet (P10), and supernatant (S10) fractions, either on a single fraction or combinations of them, as indicated above the lanes. Reaction products were analyzed as described in the legend to Fig. 1.

To further characterize the active RTC, the PNS of SARS-CoV-infected cells was subjected to differential centrifugation. A 10,000×g supernatant fraction (S10) showed no RTC activity (Fig. 3, lane 2), but only a trace amount of the original activity was recovered in the 10,000×g pellet fraction P10 (Fig. 3, lane 4). Surprisingly, RTC activity in this P10 fraction could be largely restored by adding an aliquot of the cytoplasmic S10 fraction (Fig. 3, lane 5). An S10 fraction prepared from mock-infected cells was equally capable of restoring the RTC activity in P10, indicating that a cytoplasmic host factor was required (Fig. 3, lane 6). Routinely, about 50% of the RTC activity that was originally present in the PNS could be recovered in the P10 fraction (in assays supplemented with S10). Remarkably, virtually all replicative activity was lost, while transcription was only 2- to 3-fold decreased, in the P10 fraction depleted of the host factor (Fig. 3, lane 4). The sedimentation of the RTC activity at 10,000×g suggests that it is associated with heavy membrane structures.

### RTC activity cosediments with membrane structures

**Figure 4:**
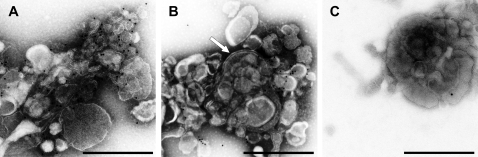
Electron micrographs of the P10 fractions of SARS-CoV-infected cells. Immunogold labeling of the P10 fraction of SARS-CoV-infected (A, B) or uninfected (C) Vero-E6 cells using rabbit antisera recognizing nsp3 (A, C) or nsp4 (B) was followed by negative staining. The white arrow indicates a part of the specimen clearly showing double membranes. Scale bar: 500 nm.

The P10 fraction, which contained the bulk of RTC activity, was analyzed by electron microscopy (negative staining) in combination with an immunogold labeling for the (putative) transmembrane proteins nsp3, nsp4, and nsp6 (Fig. 4 and data not shown). Clusters of vesicles (with diameters between 100 and 350 nm) were observed, which appeared to be associated with more tubular or flattened membrane structures. A strong immunolabeling of these structures for SARS-CoV nsp3 (Fig. 4A) and nsp4 (Fig. 4B) was observed. Membrane structures immunoreactive for nsp3 (Fig. 4C) or nsp4 (data not shown) were not detected in a control P10 fraction prepared from mock-infected cells. Occasionally, double membranes could be distinguished (Fig. 4B, arrow). These observations are consistent with the notion that the P10 fraction is enriched for SARS-CoV-induced nsp-containing membrane structures that have been documented in infected cells.

### Product-specific differences in the release of SARS-CoV RNAs from the RTC

**Figure 5:**
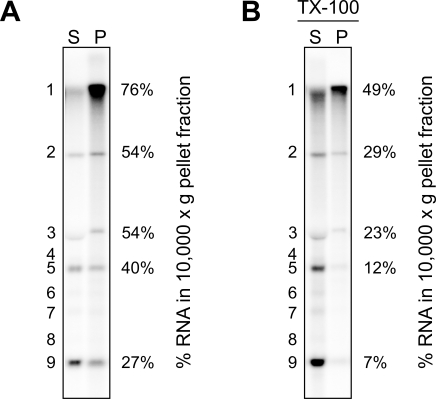
Distribution of newly synthesized SARS-CoV RNA between the RTC-containing P10 and the cytoplasmic S10 fraction. After an IVRA with the PNS from SARS-CoV infected cells, 0.5% TX-100 (B) or no detergent (A) was added and the samples were fractionated into S10 and P10 fractions. Equivalent amounts of S10 and P10 (indicated with S and P above the lanes) derived from the same number of cells were analyzed. The levels of the five most abundant RNAs in P10 and S10 fractions were quantified and for each RNA molecule the percentage that is present in P10 is indicated to the right of the gel. The signals for RNA4 and RNA6-8 were too low to be quantified reliably.

The distribution of newly synthesized SARS-CoV RNAs between the RTC-containing P10 and cytoplasmic S10 fractions was analyzed by fractionation of PNS after an IVRA (Fig. 5A). The bulk (76%) of newly made genomic RNA was recovered from the P10 fraction, suggesting it remained associated with the heavy membrane structures. In contrast, newly synthesized sg RNAs were, depending on their size, progressively more abundant in S10, suggesting their release from the RTC. To further investigate the role of membranes in RNA localization, an IVRA was performed with PNS, after which 0.5% TX-100 was added and the distribution of viral RNAs between P10 and S10 was analyzed (Fig. 5B). The bulk of the smaller RNA species (RNA5-9) was now recovered from the S10 fraction. In contrast, one-half of the genomic RNA remained associated with the P10 fraction after detergent treatment, suggesting product-specific differences in RTC operation and organization, which appears to include partly detergent-resistant structures.

### Replicase subunits co-sedimenting with the membrane-associated RTC

**Figure 6:**
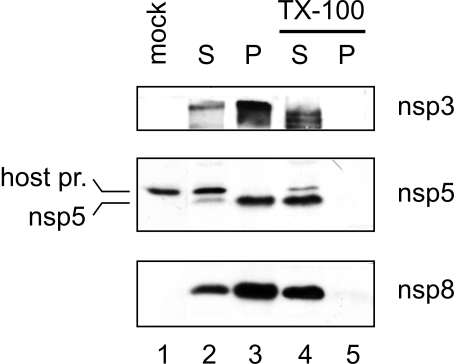
Distribution of SARS-CoV nsps between the cytoplasmic S10 fraction and RTC-containing P10 fraction. PNS from SARS-CoV-infected cells was either untreated (lanes 2 & 3) or treated with 0.5% TX-100 (lanes 4 & 5) after which it was fractionated into a 10,000×g supernatant (S) and pellet (P). Equivalent amounts of S10 and P10 derived from the same number of cells were analyzed by Western blotting with antisera recognizing nsp3, nsp5, and nsp8 antisera. Lane 1 contains the PNS from mock-infected cells. The position of a cross-reacting host protein that was recognized by the nsp5 antiserum is indicated by “host pr.” to the left of the center panel.

For selected nsps, for which suitable antisera that are reactive in Western blot experiments were available, the distribution between the cytoplasmic S10 fraction and the RTC-containing P10 fraction was analyzed. This revealed that these RTC subunits were enriched or mainly present in the P10 fraction (Fig. 6). The bulk of nsp3 was in the P10 fraction and nsp5 was found almost exclusively in the P10 fraction (Fig. 6, lane 3). Most of nsp8 was detected in the P10 fraction although also a substantial amount was found in the cytoplasmic fraction (Fig. 6, lane 2 & 3). Treatment of PNS with 0.5% TX-100 prior to P10-S10 fractionation, led to the redistribution of nsp3, nsp5, and nsp8, which were no longer found in the P10 fraction, but were recovered at increased levels in the S10 fraction (Fig. 6, lanes 4 & 5). This suggests that their direct or indirect association with membranes caused them to cosediment with the RTC activity in the P10 fraction.

### SARS-CoV nascent RNA and replicase subunits reside in a membrane-protected RTC

**Figure 7:**
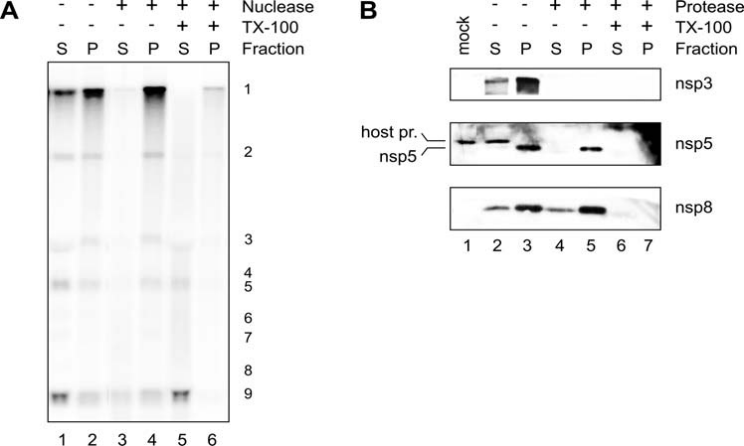
Protection of newly synthesized SARS-CoV RNA and nsps by membranes. (A) After a standard 100-min IVRA, PNS was either directly fractionated into a 10,000g pellet (lane2) and supernatant (lane 1) or treated, prior to fractionation, with nuclease Bal31 (lanes 3-6) in the presence (lanes 5 & 6) and absence (lanes 3 & 4) of 0.5% TX-100. The presence of *in vitro* synthesized radiolabeled viral RNA in the fractions was analyzed as described in the legend of Fig. 5. (B) PNS was directly fractionated into a 10,000g pellet (lane3) and supernatant (lane 2) or treated with proteinase K (lanes 4–7) in the presence (lanes 6 & 7) and absence (lanes 4 & 5) of 0.5% TX-100 prior to fractionation. The presence of nsp3, nsp5 and nsp8 in these fractions was analyzed by Western blotting, as described in the legend of Fig. 6. Lane 1 contains the PNS from mock-infected cells. The position of a host protein cross-reacting with the nsp5 antiserum is indicated by “host pr.” to the left of the blot.

To further assess the role of membranes in SARS-CoV RNA synthesis, it was investigated whether they protect the RTC. A standard 100-min IVRA was performed, followed by treatment with the nuclease Bal31, a non-specific nuclease that degrades both single- and double-stranded RNA, in the presence or absence of 0.5% TX-100. After fractionation of the samples into P10 and S10, the quantity of *in vitro* synthesized radiolabeled RNA in each fraction was analyzed (Fig. 7A). In untreated control samples, newly made viral RNA was found both associated with the RTC in the P10 fraction (predominantly genomic RNA) as well as released in the cytoplasmic S10 fraction (enriched in sg RNA; Fig 7A, lane 1 & 2). The newly made viral RNA in the cytoplasm was completely degraded upon nuclease treatment (Fig 7A, lane 3), while the RNA associated with the RTC was protected (Fig 7A, lane 4). The latter products only became susceptible to nuclease treatment upon addition of 0.5% TX-100, suggesting that the replicating RNA is enclosed by membranes (Fig 7A, compare lanes 4 and 6).

To determine whether also replicase subunits were protected by membranes, PNS was treated with proteinase K, either in the absence or presence of 0.5% TX-100. Protease-treated samples and untreated controls were subsequently fractionated into P10 and S10, after which the presence of nsp3, nsp5, and nsp8 was probed by Western blotting (Fig. 7B). Both cytoplasmic nsp3 in S10 and pelleted nsp3 in P10 were susceptible to protease treatment (Fig. 7B, top panel). The nsp5 subunit, which mainly cosedimented with the RTC in P10, was largely resistant to protease treatment (Fig 7B, middle panel, lane 5), but it was degraded in the presence of TX-100. The observed protease-resistance of nsp5 is not due to a lack of proteinase K activity, since both nsp3 and a host protein cross-reacting with the nsp5 antiserum were completely degraded in this same sample. Likewise, nsp8 in the P10 fraction was resistant to protease treatment, and, surprisingly, this was also true for the nsp8 that was present in the S10 fraction (Fig. 7B, lower panel). Both forms of nsp8 were susceptible to the protease in the presence of a non-ionic detergent (TX-100). These data suggest that nsp5 and nsp8 were enclosed by membranes. In agreement with the membrane topology predictions for the nsp3 domains used to raise our antiserum [5],[23], a major part of nsp3 was exposed on the surface of these membrane structures.

## Discussion

The SARS-CoV RTC, like the RTCs of other +RNA viruses [31]–[33], is believed to be associated with virus-induced structures derived from intracellular membranes. The coronavirus RTC is composed of an unusually large number of subunits, including several nsps with unique enzyme functions [2],[34]. Despite steady progress, the functional characterization of the 16 SARS-CoV nsps, including the RdRp and helicase enzymes that are central to replication, is still in an early stage. To investigate the details of the molecular interplay between these subunits, the viral RNA template, and host factors, *in vitro* assays for viral RNA synthesis will be indispensable. By now, the soluble expression and purification of several individual coronavirus nsps has proven to be problematic. In combination with the membrane-associated nature of the complex, this suggests that the reconstitution of the RTC from its purified components, remains a distant perspective. As a complementary approach, we therefore set out to isolate the active SARS-CoV RTC from the only currently available source: virus-infected cells. The newly developed IVRA described in this paper (Fig. 1) will allow us to obtain more insight into the architecture and function of the SARS-CoV RTC as a whole, and may aid to address the poorly defined role of cellular membranes.

Although RdRp activity in cell lysates was previously reported for the coronaviruses mouse hepatitis virus and transmissible gastroenteritis virus [35]–[40], this is to our knowledge the first description of a robust *in vitro* system for coronavirus RNA synthesis that produces the full spectrum of viral mRNAs (both genomic and sg RNAs) generated in infected cells. A similar *in vitro* system was recently developed for the distantly related arterivirus equine arteritis virus (manuscript in preparation), suggesting that our method may be generally applicable to nidovirus RTCs.

Protein synthesis occurred in our lysates under the IVRA conditions used, but its inhibition did not affect *in vitro* RTC activity (Fig. 2). This suggests that, in contrast to what was described for cells infected with mouse hepatitis virus [41]–[43] or SARS-CoV (our unpublished data), continued translation is not required for RTC activity *in vitro*. Likely, inhibition of protein synthesis does not influence the activity of the preformed, active RTCs present in our PNS, which are mainly synthesizing RNA of positive polarity (Fig. 1E).

Currently, suitable small SARS-CoV RNA replicons, which could be added to an IVRA as exogenous template and be distinguished from natural viral RNAs on the basis of size, are not available. Consequently, addressing the question whether *de novo* initiation of RNA synthesis occurs in our system must wait until further technical advances (in this area) have been made. Still, a potential complication may be the inability of such exogenous templates to enter the membrane-protected RTC, as also observed in this study for molecules like Bal31 nuclease and proteinase K (Fig. 7).

SARS-CoV RTC activity was recovered in a 10,000×g heavy membrane pellet (P10), but the isolated RTCs had to be supplemented with an S10 fraction from infected or uninfected cells to regain activity (Fig. 3). This indicates that, besides the host factors possibly associated with the RTC in the P10 fraction, also a cytoplasmic host factor is required for SARS-CoV RNA synthesis. The nature of this host factor is currently being analyzed. Replication appeared to be particularly dependent on the presence of this host factor. While transcription was only 2- to 3-fold reduced, replication was barely detectable in the P10 fraction depleted of the host factor (Fig. 3). Whether this difference is merely due to the larger size of the genomic RNA and/or reflects a higher demand or specific role for the host factor in replication remains to be investigated in more detail. The RTC activity cosedimented with newly synthesized viral RNA, several replicase subunits and nsp3-, nsp4- and nsp6-containing membrane structures. The latter proteins are (putative) multi-spanning transmembrane proteins [23],[24],[44] presumed to be important in the induction of the RTC-related membrane rearrangements that accompany SARS-CoV infection [26],[27]. Furthermore, the cosedimentation of nsp3 with the RTC (Fig. 6) may indicate that one or multiple of the enzymatic activities of this multidomain protein [5] are important for RNA synthesis. All of the nsp5 main proteinase copurified with RTC activity in the P10 fraction, although it remains to be investigated whether this finding is directly related to the site of replicase polyprotein processing. The RTC's core enzyme, the nsp12-RdRp, has been postulated to work in concert with a unique second RdRp activity that was recently identified in nsp8. Its proposed RNA primase activity [15],[45] would be consistent with the (partial) cosedimentation of nsp8 with the RTC that we observed in this study (Fig. 6). After TX-100 treatment, nsp3, nsp5, and nsp8 no longer cosedimented in P10, suggesting they had been released from the membrane structures. In addition, protease protection experiments in the absence and presence of detergents revealed that nsp5 and (part of) nsp8 were shielded by membranes, while the predicted cytoplasmic domains of nsp3 were not [23]. The experiments in Fig. 7 indicated that a cytoplasmic form of nsp8 (in S10) was also shielded from protease activity by membranes. This suggests the existence of membrane structures distinct from the RTC-containing complexes in P10. The cosedimentation of nsp3, nsp5, and nsp8 with RTC activity is in line with their colocalization in specific structures in the perinuclear region of SARS-CoV-infected cells, as observed by immunofluorescence microscopy [25],[26].

Free RNA of transmissible gastroenteritis virus was previously found to be susceptible to nuclease treatment, whereas most negative-stranded RNAs, and a small fraction of (probably nascent) positive-stranded RNAs, were present in membrane-protected complexes [46]. In our study, non-ionic detergents rendered SARS-CoV RNA susceptible to nuclease digestion (Fig. 7) and destroyed all RTC activity (Fig. 1). This again signifies the importance of intact membrane structures for viral RNA synthesis. Their disruption may have dissociated the active enzyme complex and/or changed the RTC's microenvironment, or may have provided access to cytoplasmic nucleases.

The bulk of newly synthesized SARS-CoV genome remained associated with the RTC-containing heavy membrane structures, while sg RNAs appeared to be more readily released from the structures in which they had been synthesized. In previous studies with transmissible gastroenteritis virus, it was also found that preferentially sg RNAs were no longer associated with the membrane-associated complexes [46]. The released RNA molecules might represent a pool of mRNAs destined for translation into structural and accessory proteins (sg RNAs) and additional replicase proteins (RNA1), while the RTC-associated RNAs might be engaged in replication and/or packaging. In this manner, the intracellular compartmentalization mediated by the formation of specialized membrane structures might also serve to coordinate different steps in the viral life cycle and/or enhance their specificity for viral RNA. Surprisingly, after treatment with 0.5% TX-100, a large fraction of genomic RNA remained in the 10,000×g pellet, suggesting it is associated with detergent-resistant structures. This might indicate that, as postulated for hepatitis C virus replication complexes [47]–[49], the SARS-CoV RTC is associated with lipid rafts or lipid droplets, a feature that could also explain the proposed role of lipid rafts during the early stages of SARS-CoV replication [50].

If SARS-CoV RTCs, as this study suggests, are enclosed by membranes that may provide an optimal environment for viral RNA synthesis, this raises the question of how newly synthesized RNA products are released from these structures. Moreover, the fact that RTC activity depends on a cytoplasmic host factor that does not cosediment with the complex is an additional indication that crosstalk between cytoplasm and RTC-containing membrane structures must occur, e.g. via channels that may facilitate transport across membranes. Taken together, our data support the existence of a functional link between SARS-CoV RNA synthesis and the unusual membrane structures induced upon coronavirus infection.

## Materials and Methods

### Cells, virus and antisera

Vero-E6 cells were infected with SARS-CoV (strain Frankfurt 1) at a multiplicity of infection of 5 as described previously [26]. All procedures involving live SARS-CoV were performed in the biosafety level 3 facility at Leiden University Medical Center. Rabbit antisera recognizing nsp3, nsp5, and nsp8 were described previously [26]. Antisera against nsp4 and nsp6 were raised in New Zealand White rabbits using as antigens the bovine serum albumin-coupled synthetic peptides FSNSGADVLYQPPQTSITSAVLQ and LNIKLLGIGGKPCIKVATVQ, representing the C-terminal sequences of nsp4 and nsp6, respectively.

### Isolation of enzymatically active RTCs by subcellular fractionation of SARS-CoV-infected cells

SARS-CoV- or mock-infected cells (eight 175 cm^2^ flasks) were harvested by trypsinization at 10 hours post infection. To inhibit cellular transcription, 2 µg/ml actinomycin D was present in all solutions used for harvesting and washing of the cells. After washing with PBS, cells were resuspended in 2 ml ice-cold hypotonic buffer (20 mM HEPES, 10 mM KCl, 1.5 mM MgOAc_2_, 1 mM DTT, 133 U/ml RNaseOUT (Invitrogen) and 2 µg/ml actinomycin D, pH 7.4) and incubated for 10 min at 4°C. Cells were disrupted using a Dounce homogenizer by giving 30 strokes with a tight fitting pestle. Isotonic conditions were restored by adding HEPES, sucrose, and DTT, which resulted in a final lysate containing 35 mM HEPES, pH 7.4, 250 mM sucrose, 8 mM KCl, 2.5 mM DTT, 1 mM MgOAc_2_, 2 µg/ml actinomycin D, and 130 U/ml RNaseOUT. Nuclei, large debris, and any remaining intact cells were removed by two successive 5-min centrifugations at 1,000×g, and the resulting PNS was either assayed immediately for RTC activity or stored at −80°C. The SARS-CoV titer present in PNS was approximately 10^8^ plaque-forming units per ml. Plaque assays performed before and after IVRAs revealed that no measurable *de novo* virus production occurred during this assay (data not shown). A 10,000×g pellet (P10) and supernatant (S10) fraction were prepared from PNS by centrifugation at 10,000×g for 10 min. The pellet was resuspended in dilution buffer (35 mM HEPES, 250 mM sucrose, 8 mM KCl, 2.5 mM DTT, 1 mM MgOAc_2_, pH 7.4), in 1/10 of the original PNS volume from which the pellet had been prepared. In some experiments, PNS was incubated for 15 min at 4°C with 0.5% TX-100 prior to the preparation of P10 and S10 fractions.

### *In vitro* RNA synthesis assay (IVRA)

Assays were performed using either 25 µl PNS, 20 µl S10, 5 µl P10, or 5 µl P10 supplemented with 20 µl S10. When required, the total volume was adjusted to 25 µl with dilution buffer. The subsequent addition of reaction components yielded a 28 µl final reaction volume, containing 30 mM HEPES pH 7.4, 220 mM sucrose, 7 mM KCl, 2.5 mM DTT, 2 mM MgOAc_2_, 2 µg/ml actinomycin D, 25 U RNaseOUT, 20 mM creatine phosphate (Sigma), 10 U/ml creatine phosphokinase (Sigma), 1 mM ATP, 0.25 mM GTP, 0.25 mM UTP, 0.6 µM CTP and 0.12 µM and 10 µCi [α-^32^P]CTP (GE Healthcare). Unless otherwise indicated, IVRAs were performed for 100 min at 30°C. Reactions were terminated by adding 60 µl of a mixture containing 5% lithium dodecyl sulfate, 0.1 M Tris-HCl, pH 8.0, 0.5 M LiCl, 10 mM EDTA, 5 mM DTT, and 0.1 mg/ml proteinase K, and incubating at 37°C for 10 min. When protein synthesis was tested, [α-^32^P]CTP was replaced with 14.3 µCi of Promix (GE Healthcare), containing a mixture of [^35^S]methionine and [^35^S]cysteine. To assess the effect of translation inhibition, 70 µg/ml of cycloheximide or 350 µg/ml of puromycin were added.

### RNA Isolation and analysis

RNA was isolated from IVRA reaction mixtures by acid phenol extraction and isopropanol precipitation. Reaction products were analyzed by denaturing formaldehyde agarose gel electrophoresis essentially as described previously, except that a 1% agarose gel was used [51]. Radiolabeled *in vitro* synthesized RNA was detected by exposing a PhosphorImager screen directly to the dried gel, after which screens were scanned with a Personal Molecular Imager FX (Bio-Rad) and data were analyzed with Quantity One version 4.5.1 (Bio-Rad). Unlabeled endogenous SARS-CoV RNA was detected by hybridization with a ^32^P-labeled oligonucleotide SARSV002 (5′-CACATGGGGATAGCACTAC-3′), which is complementary to a sequence present in the 3′-end of all SARS-CoV RNAs [5].

### Determination of the polarity of *in vitro* synthesized SARS-CoV RNA

*In vitro* transcribed RNAs (0.75 µg) corresponding to nt 29,364-29,727 of the 3′-terminal region (3′-TR(+)) or complementary to nt 1-378 (3′-TR(−)) of the SARS-CoV genome were immobilized to Hybond N+ membrane (GE Healthcare). As negative controls, RNAs corresponding to nt 12,313–12,660 (ctrl. a) of the equine arteritis virus genome or its complementary sequence (ctrl. b) were included. The membrane with the immobilized probes was prehybridized for 4 hours in a hybridization mixture containing 5×SSPE (750 mM NaCl, 50 mM NaH_2_PO_4_, 5 mM EDTA, pH 7.0), 0.05% SDS, 5x Denhardt and 100 µg/ml homomix I. Subsequently, the membrane was hybridized with half of the ^32^P-labeled RNA recovered from a 28-µl IVRA in 0.8 ml hybridization mix, which was first heat denatured at 70°C for 15 min. After hybridization for 16 h at 56°C, membranes were washed twice for 20 min at 56°C with 4 ml of 5x SSPE, 0.05% SDS, and the hybridization signal was quantified by PhosphorImager analysis.

### SDS-PAGE and Western blotting

Proteins were separated by SDS-PAGE and transferred to Hybond-P PVDF membrane (GE Healthcare) by semi-dry blotting. After blocking with 1% casein in PBS containing 0.1% Tween-20 (PBST), membranes were incubated with anti-nsp3, anti-nsp5 or anti-nsp8 rabbit antisera, diluted 1∶2000 in PBST with 0.5% casein and 0.1% BSA. Peroxidase-conjugated swine anti-rabbit IgG antibody (DAKO) and the ECL-plus kit (GE Healthcare) were used for detection.

### Protease and nuclease protection assays

Protease protection experiments were done by incubating PNS (50 µl) for 10 min at 20°C with 20 µg/ml of proteinase K either in the absence or presence of 0.5% TX-100. After inactivation of the protease by addition of 2 mM PMSF and fractionation into a 10,000×g pellet (P10) and supernatant (S10), samples were analyzed by Western blotting. For nuclease protection assays, a standard 100-min IVRA was performed with the PNS, after which 5U of Bal31 nuclease was added, either in the presence or in the absence of 0.5% TX-100. After a 10-min incubation, samples were fractionated into S10 and P10 fractions. Radiolabeled RNA was isolated from the fractions and analyzed as described above.

### Electron microscopy

One volume of 6% paraformaldehyde in 60 mM PIPES, 25 mM HEPES, 2 mM MgCl_2_, 10 mM EGTA, pH 6.9 was added to P10 fractions. Formvar-coated grids were placed on 10-µl drops of these fixed P10 fractions and incubated at room temperature for 1 min. After blocking with 1% BSA in PBS, grids were incubated for 30 min with rabbit antisera directed against nsp3, nsp4 or nsp6 (1∶200) in PBS containing 1% BSA. Bound rabbit IgG was detected with protein A carrying 15-nm gold particles. After negative staining with 2% phosphotungstic acid, grids were viewed in a FEI T12 transmission electron microscope at 120 kV.

## References

[1] Peiris JS, Guan Y, Yuen KY (2004). Severe acute respiratory syndrome.. Nat Med.

[2] Gorbalenya AE, Enjuanes L, Ziebuhr J, Snijder EJ (2006). Nidovirales: evolving the largest RNA virus genome.. Virus Res.

[3] Pasternak AO, Spaan WJ, Snijder EJ (2006). Nidovirus transcription: how to make sense...?. J Gen Virol.

[4] Sawicki SG, Sawicki DL, Siddell SG (2007). A contemporary view of coronavirus transcription.. J Virol.

[5] Snijder EJ, Bredenbeek PJ, Dobbe JC, Thiel V, Ziebuhr J (2003). Unique and conserved features of genome and proteome of SARS-coronavirus, an early split-off from the coronavirus group 2 lineage.. J Mol Biol.

[6] Thiel V, Ivanov KA, Putics A, Hertzig T, Schelle B (2003). Mechanisms and enzymes involved in SARS coronavirus genome expression.. J Gen Virol.

[7] Lindner HA, Fotouhi-Ardakani N, Lytvyn V, Lachance P, Sulea T (2005). The papain-like protease from the severe acute respiratory syndrome coronavirus is a deubiquitinating enzyme.. J Virol.

[8] Harcourt BH, Jukneliene D, Kanjanahaluethai A, Bechill J, Severson KM (2004). Identification of severe acute respiratory syndrome coronavirus replicase products and characterization of papain-like protease activity.. J Virol.

[9] Yang H, Yang M, Ding Y, Liu Y, Lou Z (2003). The crystal structures of severe acute respiratory syndrome virus main protease and its complex with an inhibitor.. Proc Natl Acad Sci U S A.

[10] Graziano V, McGrath WJ, DeGruccio AM, Dunn JJ, Mangel WF (2006). Enzymatic activity of the SARS coronavirus main proteinase dimer.. FEBS Lett.

[11] Tan YJ, Lim SG, Hong W (2005). Characterization of viral proteins encoded by the SARS-coronavirus genome.. Antiviral Res.

[12] Ziebuhr J (2004). Molecular biology of severe acute respiratory syndrome coronavirus.. Curr Opin Microbiol.

[13] Cheng A, Zhang W, Xie Y, Jiang W, Arnold E (2005). Expression, purification, and characterization of SARS coronavirus RNA polymerase.. Virology.

[14] Ivanov KA, Thiel V, Dobbe JC, van der Meer Y, Snijder EJ (2004). Multiple enzymatic activities associated with severe acute respiratory syndrome coronavirus helicase.. J Virol.

[15] Imbert I, Guillemot JC, Bourhis JM, Bussetta C, Coutard B (2006). A second, non-canonical RNA-dependent RNA polymerase in SARS coronavirus.. EMBO J.

[16] Zhai Y, Sun F, Li X, Pang H, Xu X (2005). Insights into SARS-CoV transcription and replication from the structure of the nsp7-nsp8 hexadecamer.. Nat Struct Mol Biol.

[17] Minskaia E, Hertzig T, Gorbalenya AE, Campanacci V, Cambillau C (2006). Discovery of an RNA virus 3′->5′ exoribonuclease that is critically involved in coronavirus RNA synthesis.. Proc Natl Acad Sci U S A.

[18] Eckerle LD, Lu X, Sperry SM, Choi L, Denison MR (2007). High fidelity of murine hepatitis virus replication is decreased in nsp14 exoribonuclease mutants.. J Virol.

[19] Bhardwaj K, Sun J, Holzenburg A, Guarino LA, Kao CC (2006). RNA recognition and cleavage by the SARS coronavirus endoribonuclease.. J Mol Biol.

[20] Ivanov KA, Hertzig T, Rozanov M, Bayer S, Thiel V (2004). Major genetic marker of nidoviruses encodes a replicative endoribonuclease.. Proc Natl Acad Sci U S A.

[21] Bhardwaj K, Guarino L, Kao CC (2004). The severe acute respiratory syndrome coronavirus Nsp15 protein is an endoribonuclease that prefers manganese as a cofactor.. J Virol.

[22] Ricagno S, Egloff MP, Ulferts R, Coutard B, Nurizzo D (2006). Crystal structure and mechanistic determinants of SARS coronavirus nonstructural protein 15 define an endoribonuclease family.. Proc Natl Acad Sci U S A.

[23] Kanjanahaluethai A, Chen Z, Jukneliene D, Baker SC (2007). Membrane topology of murine coronavirus replicase nonstructural protein 3.. Virology.

[24] Oostra M, te Lintelo EG, Deijs M, Verheije MH, Rottier PJ (2007). Localization and membrane topology of coronavirus nonstructural protein 4: involvement of the early secretory pathway in replication.. J Virol.

[25] Prentice E, McAuliffe J, Lu X, Subbarao K, Denison MR (2004). Identification and characterization of severe acute respiratory syndrome coronavirus replicase proteins.. J Virol.

[26] Snijder EJ, van der Meer Y, Zevenhoven-Dobbe J, Onderwater JJ, van der Meulen J (2006). Ultrastructure and origin of membrane vesicles associated with the severe acute respiratory syndrome coronavirus replication complex.. J Virol.

[27] Stertz S, Reichelt M, Spiegel M, Kuri T, Martinez-Sobrido L (2007). The intracellular sites of early replication and budding of SARS-coronavirus.. Virology.

[28] Goldsmith CS, Tatti KM, Ksiazek TG, Rollin PE, Comer JA (2004). Ultrastructural characterization of SARS coronavirus.. Emerg Infect Dis.

[29] Ng ML, Tan SH, See EE, Ooi EE, Ling AE (2003). Proliferative growth of SARS coronavirus in Vero E6 cells.. J Gen Virol.

[30] Kamitani W, Narayanan K, Huang C, Lokugamage K, Ikegami T (2006). Severe acute respiratory syndrome coronavirus nsp1 protein suppresses host gene expression by promoting host mRNA degradation.. Proc Natl Acad Sci U S A.

[31] Mackenzie J (2005). Wrapping things up about virus RNA replication.. Traffic.

[32] Salonen A, Ahola T, Kaariainen L (2005). Viral RNA replication in association with cellular membranes.. Curr Top Microbiol Immunol.

[33] Novoa RR, Calderita G, Arranz R, Fontana J, Granzow H (2005). Virus factories: associations of cell organelles for viral replication and morphogenesis.. Biol of the Cell.

[34] Ziebuhr J (2005). The coronavirus replicase.. Curr Top Microbiol Immunol.

[35] Brayton PR, Lai MM, Patton CD, Stohlman SA (1982). Characterization of two RNA polymerase activities induced by mouse hepatitis virus.. J Virol.

[36] Brayton PR, Stohlman SA, Lai MM (1984). Further characterization of mouse hepatitis virus RNA-dependent RNA polymerases.. Virology.

[37] Mahy BW, Siddell S, Wege H, ter MV (1983). RNA-dependent RNA polymerase activity in murine coronavirus-infected cells.. J Gen Virol.

[38] Compton SR, Rogers DB, Holmes KV, Fertsch D, Remenick J (1987). In vitro replication of mouse hepatitis virus strain A59.. J Virol.

[39] Dennis DE, Brian DA (1982). RNA-dependent RNA polymerase activity in coronavirus- infected cells.. J Virol.

[40] Leibowitz JL, DeVries JR (1988). Synthesis of virus-specific RNA in permeabilized murine coronavirus-infected cells.. Virology.

[41] Sawicki SG, Sawicki DL (1986). Coronavirus minus-strand RNA synthesis and effect of cycloheximide on coronavirus RNA synthesis.. J Virol.

[42] Baric RS, Yount B (2000). Subgenomic negative-strand RNA function during mouse hepatitis virus infection.. J Virol.

[43] Kim JC, Spence RA, Currier PF, Lu X, Denison MR (1995). Coronavirus protein processing and RNA synthesis is inhibited by the cysteine proteinase inhibitor E64d.. Virology.

[44] Sparks JS, Lu X, Denison MR (2007). Genetic analysis of Murine hepatitis virus nsp4 in virus replication.. J Virol.

[45] Sutton G, Fry E, Carter L, Sainsbury S, Walter T (2004). The nsp9 replicase protein of SARS-coronavirus, structure and functional insights.. Structure.

[46] Sethna PB, Brian DA (1997). Coronavirus genomic and subgenomic minus-strand RNAs copartition in membrane-protected replication complexes.. J Virol.

[47] Aizaki H, Lee KJ, Sung VM, Ishiko H, Lai MM (2004). Characterization of the hepatitis C virus RNA replication complex associated with lipid rafts.. Virology.

[48] Shi ST, Lee KJ, Aizaki H, Hwang SB, Lai MM (2003). Hepatitis C virus RNA replication occurs on a detergent-resistant membrane that cofractionates with caveolin-2.. J Virol.

[49] Miyanari Y, Atsuzawa K, Usuda N, Watashi K, Hishiki T (2007). The lipid droplet is an important organelle for hepatitis C virus production.. Nat Cell Biol.

[50] Li GM, Li YG, Yamate M, Li SM, Ikuta K (2007). Lipid rafts play an important role in the early stage of severe acute respiratory syndrome-coronavirus life cycle.. Microbes Infect.

[51] van Marle G, van Dinten LC, Spaan WJ, Luytjes W, Snijder EJ (1999). Characterization of an equine arteritis virus replicase mutant defective in subgenomic mRNA synthesis.. J Virol.

